# Use of smartphone and social media under 15 year should be forbidden/regulated Con Speaker

**DOI:** 10.1192/j.eurpsy.2025.38

**Published:** 2025-08-26

**Authors:** T. van Amelsvoort

**Affiliations:** Psychiatry & Neuropsychology, Maastricht University, Maastricht, Netherlands

## Abstract

**Abstract:**

Worldwide, social media and touch screen device use has rapidly increased over the past decade and are particularly popular among young people. To obtain more insight in the potential negative associations with compulsive social media use and touch screen devices in Dutch children, we assessed its relation with self-reported well-being and cognitive function in primary school children. We found social media to be negatively associated with life satisfaction and increasing touch screen device use with increased reaction time. This suggests that the compulsive social media use and touch screen device use have negative consequences on mental well being and cognitive performance already at a young age. Therefore the use of social media in children under the age of 15 years should be restricted or forbidden.

**Disclosure of Interest:**

None Declared

